# Patients' Voices and Dietitians' Perspectives on Meaningful Aspects in the Nutritional Care of Patients at Risk of Malnutrition After Stroke

**DOI:** 10.1111/jhn.70091

**Published:** 2025-07-28

**Authors:** Jenny McGreevy, Anne‐Marie Boström, Lena Nordgren, Ylva Orrevall, Elin Lövestam

**Affiliations:** ^1^ Department of Food Studies, Nutrition and Dietetics Uppsala University Uppsala Sweden; ^2^ Centre for Clinical Research Sörmland Uppsala University Uppsala Sweden; ^3^ Department of Dietetics Nykoping Hospital Nykoping Sweden; ^4^ Department of Neurobiology, Care Science and Society Division of Nursing, Karolinska Institutet Huddinge Sweden; ^5^ Theme Inflammation and Aging Karolinska University Hospital Huddinge Sweden; ^6^ Research and Development Unit, Stockholms Sjukhem Stockholm Sweden; ^7^ Department of Public Health and Caring Sciences Uppsala University Uppsala Sweden; ^8^ Medical Unit Allied Health Professionals, Women's Health and Allied Health Professionals Theme Karolinska University Hospital Stockholm Sweden

**Keywords:** altered eating, dietitian, malnutrition, outcome measures, patient experiences, stroke, tools

## Abstract

**Background:**

After a stroke, patients may have complex eating difficulties that can lead to a risk for malnutrition. Individualised nutritional care could be optimised by identifying meaningful factors in the patient's relationship with food and eating. This study explored the perspectives of dietitians regarding the nutritional care of these patients and the experiences of patients in relation to food and eating in everyday life at home.

**Methods:**

Two focus groups with five registered dietitians in each and eight individual semi‐structured patient interviews were conducted. The two data sets were analysed separately with inductive qualitative analysis using a thematic analysis approach.

**Results:**

In the nutritional care of these patients, the dietitians specifically highlighted: (1) *Practical aspects,* (2) *Support and social aspects* and (3) *Feelings and emotions*. The patient interviews revealed four themes: (1) *Ability to prepare and eat food*, (2) *Issues affecting food enjoyment*, (3) *Social aspects of eating and mealtimes* and (4) *Emotional relationship with food*.

**Conclusions:**

Patients described symptoms after a stroke impacting food enjoyment, social identity, and well‐being. While dietitians reported addressing practical, social, and emotional aspects of food and eating, they lacked appropriate tools to measure these. There was a discrepancy between the focus of dietitians and that of patients; appropriate tools are needed to address these perspectives to provide high‐quality patient‐centred care.

## Summary

- Dietitians evaluate the nutritional care of patients at risk of malnutrition after a stroke using measures of body weight and intake, and while social and emotional aspects are described as important, appropriate tools to assess and evaluate these are lacking.
- Patients describe how the negative impact of symptoms after a stroke profoundly impact their everyday life in relation to food and eating.
- The burden to patients of the profound negative impact of their changed relationship to food and eating after a stroke are not fully captured by outcome measures currently available to dietitians.

## 1 Introduction

Malnutrition can be defined as ‘*a state resulting from a lack of intake or uptake of nutrition that leads to altered body composition (decreased fat free mass) and body cell mass leading to diminished physical and mental function and impaired clinical outcome from disease*’ [1]. It is estimated to occur in about a quarter of people who have a stroke during the early stages of care [2, 3], increasing the risk of infection, extended hospital stays, and mortality [4]. These patients can be particularly at risk of malnutrition due to eating difficulties that may be complex, requiring multiple strategies to ensure that their nutritional needs are met and facilitate rehabilitation [5, 6]. For some, eating difficulties may become permanent, leading to a risk of developing malnutrition during and after rehabilitation [4, 5, 7, 8]. Studies have shown that eating difficulties following a stroke and malnutrition may lead to impaired quality of life [9, 10, 11, 12].

The dietitian's role in the nutritional care of these patients is to investigate, assess and diagnose nutritional problems, and to implement, monitor and evaluate individualised interventions [5, 13]. The Nutrition Care Process (NCP), developed by the Academy of Nutrition and Dietetics, provides dietitians with a systematic framework involving four steps for nutritional care: nutrition assessment, nutrition diagnosis, nutrition intervention, and nutrition monitoring and evaluation [14]. The NCP puts the patient at the centre of the nutritional care and healthcare professionals can evaluate the care using outcomes [15]. In individualised care, or patient‐centred care (PCC), the relationship between the patient and the healthcare professional is considered prerequisite for the delivery of quality healthcare; PCC is advocated in policy documents [16, 17, 18]. However, PCC has not been clearly defined in relation to nutritional care [15, 19, 20], which can lead to inconsistencies in the way PCC is carried out and evaluated [15, 21]. The identification of factors that are meaningful for the patient can help inform the nutrition intervention and allow the evaluation of outcomes most relevant for them. These might be, for example, qualitative aspects, such as being able to participate in family life and free‐time activities, undertake activities of daily living, or be as mobile as possible [15]. The structured evaluation of treatment outcomes has been shown to increase the quality of healthcare and patient safety; however, suitable measures and tools to evaluate nutrition interventions, particularly from the patient's perspective, are lacking [22].

Previous studies in our research group have explored the process of evaluating nutrition interventions for patients at risk of malnutrition. Focus groups with dietitians revealed that dietitians reported evaluating nutritional care outcomes using mostly quantitative measures, such as body weight and nutritional intake [23]. The lack of tools to describe and follow qualitative outcomes was also highlighted. Further, individual interviews with patients with various underlying medical diagnoses at risk of malnutrition explored their experiences regarding goals, revealing discrepancies between dietitians' goals and patients' perspectives [24]. The present study focuses specifically on patients at risk of malnutrition after a stroke. The overall objective was to perform a multifaceted exploration of the perspectives of dietitians on their nutritional care of these patients and the experiences of patients regarding food, meals, and eating in their daily life at home. The knowledge gained from the present study could lead to improved individualised nutritional care of patients at risk of malnutrition after a stroke (from here on referred to as ‘this patient group’) and the future development of a measure to evaluate this care from the patient's perspective. The overarching aim is, therefore, to enhance the quality of individualised nutritional care of patients at risk of malnutrition after a stroke by exploring the perspectives of both dietitians and patients on nutritional care, food, and eating.

The specific aims were to:

- Investigate aspects that dietitians focus on during the nutritional care of this patient group;
- Investigate aspects of food and eating that this patient group describe as important for their daily living;
- Explore these aspects in relation to each other.

## 2 Methods

### 2.1 Study Design

This study used a qualitative research design with data triangulation. The starting point was our previous work on the nutritional care of patients at risk of malnutrition [23]. Data collection in the present study occurred in two distinct phases. In the first phase, we analysed data related to the nutritional care of this patient group, gathered during focus group discussions with dietitians. Although discussions were conducted during our earlier work, data specifically collected for this study had not been previously analysed.

In the second phase, semi‐structured interviews were held with individuals from this patient group living at home. Patients were invited to share their thoughts and reflect on their current situation in relation to food, mealtimes, and eating. To gain an overall understanding of the results, the data sets from the two phases were first analyzed separately.

### 2.2 Setting and Samples

In the first phase, two focus groups with registered dietitians from primary care and hospitals in central Sweden were conducted in October and November 2019. Purposive sampling was used to recruit dietitians fulfilling the following inclusion criteria:

- Currently working at least 50% of full‐time with this patient group;
- Having at least 1‐years' experience in their current role working with this patient group.

After approval from their department manager, 10 dietitians participated in two focus groups, five in each, held at the two district hospitals. The majority were female, all had a bachelor's degree and one a master's degree. Most worked with either outpatients (*n* = 5) or inpatients (*n* = 2), while others worked with both (*n* = 3). Participant characteristics are reported in Table 1.

**Table 1:** Demographics of participants in focus groups, in total 10 dietitians.

Focus group	1	2
Number of participants	5	5
Year of experience as a dietitian		
1–8		2
9–19	3	2
20–30	2	1
Highest degree		
Bachelor's exam	4	5
Master's exam	1	0
Area of clinical practice		
Only outpatients	4	1
Only inpatients	1	1
Both outpatients and inpatients		3

In the second phase, purposive sampling was used where dietitians from the same two district hospitals and regional primary care as in the first phase of this study were invited to help identify patients who could be contacted regarding participation in individual interviews. The inclusion criteria were as follows:

- Patients > 18 years.
- Clinically diagnosed stroke 3–18 months ago.
- Living in own home ≥ 1 month after stroke.
- Contact with dietitian in past month.
- Can eat and drink by mouth.
- Risk for malnutrition according to the Swedish National Board of Health and Welfare's criteria [25]:
  ▪ *Unintended weight loss.*
  ▪ *Underweight (BMI* < *20 kg/m2 at* < *70 years; BMI* < *22 kg/m^2^ at* > *70 years)*.
  ▪ *Eating difficulties.*
- Symptoms after stroke that affect food and eating.
- Assessed by dietitian to have:
  ▪ *Adequate cognitive ability.*
  ▪ *Good Swedish or English Language skills.*

Persons with good insight into the patient's everyday life could support those having difficulties speaking.

The dietitians received the inclusion criteria and information to give patients about the study in writing. Contact details for eligible patients interested in more information were sent to the first author (JM) who telephoned them. Three patients declined participation and two were not eligible. Interviews were held with eight patients, living at home, recruited from primary care and hospitals in a single healthcare region in central Sweden between 2020 and 2024. All had suffered a stroke within the past year at the time of interview. Four males and four females, aged 52–86 years (median 73.5 years), participated. Six were retired and two were on sick leave; five shared a household with others. Participant characteristics are presented in Table 2.

**Table 2:** Demographics of patients interviewed.

Females	4
Males	4
Age range	52‐86 years (median 73.5)
Employment status	
Pensioner	6
Sick leave	2
Living alone	3
Living in shared household	5

### 2.3 Data Collection

For the focus groups with dietitians, a semi‐structured interview guide (see Appendix A) was developed by the authors based on the objectives of the study. The participants were asked to share thoughts and reflect upon their nutritional care of the patient group regarding nutritional assessment, monitoring and evaluation, outcome measurement, and factors contributing to successful nutritional care. The first author (JM) acted as moderator, and a member of the research group as observer; each focus group lasted approximately 90 min. Both moderator and observer are trained in qualitative research. The discussions were conducted in Swedish, transcribed verbatim and anonymised. All participants were colleagues of, not supervised by or subordinate to, the moderator; they had not had previous contact with the observer.

Individual patient interviews were conducted by the first author with eight patients at their chosen location; all were interviewed in their own homes. A semi‐structured interview guide (see Appendix B) was developed by the authors based on the objectives of the study. Participants were invited to reflect on how they currently experienced food, meals and eating compared to before their stroke, and how their stroke had impacted their life and everyday functioning. In two interviews, a family member was present to help the participant who had difficulties speaking. The following open questions were asked:

*• How do you feel about meals, eating, and drinking after your stroke?*

*• How were meals and eating before your stroke? Did you have any problems eating before your stroke?*

*• How do you feel that changes to meals, eating and drinking after your stroke have affected your life?*

Follow‐up questions were posed to obtain more in‐depth information. The interviews, held in Swedish, lasted on average about 45 min, were audio‐recorded and transcribed verbatim; quotes presented in the results have been translated into English by the first author, a bilingual dietitian and professional translator. The participants had not had previous contact with any of the authors.

### 2.4 Data Analysis

The two data sets were analysed separately using inductive qualitative analysis described by Carolan et al. 2014 [26], which is informed by Burnard's thematic analysis approach [27]. The following steps guided the process: (1) Repeated reading of the transcripts to become immersed in the data, while writing memos about emerging ideas; (2) seeking out common concepts, ideas, and perceptions; (3) classifying the concepts into themes; (4) repeatedly reading and reviewing the themes and the data; (5) returning to the data to seek alternative meanings for the themes. The themes were then discussed by the authors and finalised. Data analysis was undertaken by hand. The recordings and the interview transcripts were referred to regularly to ensure that the analysis stayed close to the original meanings and contexts.

### 2.5 Ethical Considerations

The study was approved by the Swedish Ethical Review Authority (Dnr 2019‐0256; amended 2022‐04286‐02) and performed in accordance with the ethical standards of the 1964 Declaration of Helsinki [28]. All participants received verbal and written information about the purpose of the study, details of participation, and possibility to withdraw participation at any time, without explanation or consequences. All gave verbal and written consent to participate, including permission to audio‐record the interviews.

## 3 Results

### 3.1 Focus Groups With Dietitians

The dietitians described the specific aspects they consider during the nutritional care of patients at risk of malnutrition after a stroke regarding nutritional assessment, monitoring and evaluation, and factors contributing to successful nutritional care. The analysis identified three themes that the dietitians considered significant to these patients and important in their nutritional care: (1) *Practical aspects*, (2) *Support and social aspects* and (3) *Feelings and emotions*.

#### 3.1.1 Practical Aspects

They described the importance of assessing and following‐up a patient's access to food and their ability to shop for food, especially during the winter, as well as functional issues concerning food preparation and cooking. They would determine if the patient had difficulties eating, chewing and/or swallowing that could influence the nutritional advice regarding the consistency of foods and the patient's ability to manage any restrictions. Other factors addressed might be if a meal takes a long time to eat or if the patient becomes tired during a meal.

“Can they cook, use their hand, hold a fork, eat from a plate, cut the food, do they have neglect, swallowing problems, food sticking in the mouth, chewing difficulties, decreased appetite, consistency modification needs.”

(Group 2, person 4)

However, despite reporting a focus on practical aspects around food and eating, these were not systematically evaluated in a measurable way. The dietitians were aware that these dimensions need to be given more focus but highlighted the lack of suitable tools to enable their structured assessment, monitoring, and evaluation. This led to only quantitative measures, such as body weight and nutritional intake, being used to follow‐up and evaluate the nutritional care.

#### 3.1.2 Support and Social Aspects

Another aspect that dietitians assessed was whether the patient received assistance with food and meals and, if so, who provided this. They described asking about the patient's home situation, if they live alone, if they want or have home care services, or maybe need more. They asked if someone prepares food for them or helps with food shopping. Sometimes a spouse may have taken over the cooking with verbal instructions from the patient. All such information might influence the nutritional care and advice. The dietitians would try to address any difficulties, for example by initiating support from home care services.

“‘Maybe we should think about getting meals delivered, home care services’, it's about the food situation itself.”

(Group 1, Person 3)

Although the dietitians might evaluate a nutritional intervention based on a patient's weight, the primary goal was that food and eating should function well in the patient's everyday life. They emphasized that consideration of such aspects is important, but difficult to follow‐up in a structured and measurable way.

#### 3.1.3 Feelings and Emotional Aspects

The dietitians described trying to investigate the patient's feelings regarding their new situation in relation to food and eating, and any emotional aspects affecting food intake. They reported that some patients feel an enormous sense of loss over not being able to cook or eat the same food as before, for example needing consistency modified food and drink; however, these emotional aspects are not measured or followed‐up in a systematic way.

“It's important to ask how they feel. I met someone who loved to cook before their stroke and now felt huge sadness at not being able to stand in the kitchen making food. Now this person doesn't want to eat saying ‘why should I eat when I can't cook my own food.’”

(Group 2, person 3)

### 3.2 Patient Interviews

In the patient interviews, the participants were invited to share their thoughts and experiences regarding food, meals, and eating, reflecting on how they currently experienced these compared to before their stroke. They described aspects relating to four themes: (1) *Ability to prepare and eat food*, (2) *Issues affecting food enjoyment*, (3) *Social eating and mealtimes*, (4) *Emotional relationship with food*.

#### 3.2.1 Ability to Prepare and Eat Food

Participants reported physical limitations after their stroke that resulted in a loss of independence regarding, for example, shopping, cooking, or accessing food and drink at home. Some were no longer able to drive so relied on others to buy food or drive them to the shops. One participant who lived alone and was immobile had to wait for the home care services to arrive and fetch food or drink from the fridge, describing how sometimes they couldn't find what was wanted. Some reported difficulties preparing food or transferring food from the plate to the mouth. This could be due to impaired sight or weakness in one side of the body after the stroke, making it difficult to cut food up, eat with a knife and fork, or stand at the cooker.

“I've lost the sight in one eye due to the stroke; the left side of my body is also very weak. The preparation of food takes much longer now … I've adjusted the type of meals I eat around my physical limitations … I choose food that I can prepare with one hand.”

(Int. 6)

Participants reported symptoms after their stroke which impacted their ability to eat. One described how a lack of sensation in their face, mouth and tongue made it difficult to move food around the mouth, which also affected taste perception. Another described how some foods collected in one side of their mouth and were difficult to clear, these were therefore avoided. Others described diminished ability to chew or swallow after the stroke.

#### 3.2.2 Issues Affecting Food Enjoyment

Some participants reported reduced perceptions of taste and smell while another described a heightened sensation of certain tastes, such as spicy food and sweet things, which affected the enjoyment of food. Certain foods now tasted bland or strange so were avoided. Another described how the texture and feel of food in the mouth were now unpleasant. One participant described having previously enjoyed cooking for guests but because of taste alterations, it was now impossible to tell if the food tasted nice or not, which the participant found upsetting.

“The food that the home care service brings smells strange. It makes the meals difficult to eat, they all smell the same…. Coffee doesn't taste good anymore.”

(Int. 5)

The participants also described physical and mental fatigue after their stroke. They experienced impairment of thought processes making it difficult to concentrate and focus, affecting their ability to plan and organise food and meals. Some struggled to think clearly in noisy environments, such as shops. If they had difficulty deciding what to buy, they just bought the foods they knew others liked, which altered their enjoyment of shopping and cooking, and their role in the family.

“When I go shopping, there's a lot to think about… I have mental fatigue. I used to decide what we ate, what I fancied eating. Now I just prepare the food my husband likes.”

(Int. 4)

#### 3.2.3 Social Eating and Mealtimes

Participants described avoiding social participation after their stroke, perceiving this as a loss. This could be due to difficulties eating that might be considered socially unacceptable, such as problems getting food into their mouth, noisy eating, food dropping or hanging from their mouth, or the risk of coughing. Some had become very isolated due to not being able to drive. One reported eating meals with friends before their stroke but now only having telephone contact.

“I enjoyed meeting people when I could drive into town and eat at different restaurants. Now I'm not allowed to drive.”

(Int. 2)

Others described how their role in the family or among friends had changed, which affected the responsibilities they now had and their social identity. Some reported feeling they were viewed differently by family members because of needing help with, for example, preparing and cooking food. One participant described how the family used to come over for meals. Now they all meet elsewhere, significantly changing the participant's role and identity within the family.

“Everyone used to love my cooking. I have a big family. Now we don't all get together here anymore. That's over (becomes upset). Now we meet at someone else's place – it's not the same.”

(Int. 4)

#### 3.2.4 Emotional Relationship With Food

Almost all those interviewed reported that the pleasure of food and eating had been lost, which some found depressing. Symptoms, such as altered taste perception, being unable to shop or cook for themselves, and the sheer physical work and functional difficulties involved in eating, could contribute to this loss. Some described an indifference towards food and loss of appetite after their stroke. One saw no point in going to a restaurant anymore because they couldn't appreciate the food. Food and meals had become a need to nourish their bodies rather than something they looked forward to or found pleasurable.

“It's depressing when I can't eat what I want. Food was always important to me.”

(Int. 5)

Some talked about enjoying the food visually, thinking it looks tasty but then only eating a small amount. Others talked about enjoying the company of others at mealtimes, but not the food.

“During the week when I'm home alone… I don't want to eat. At the weekend, we all eat together… then I eat.”

(Int. 7)

## 4 Discussion

The overarching aim of this study is to enhance the quality of individualised nutritional care of patients at risk of malnutrition after a stroke by performing a multifaceted exploration of the perspectives of dietitians and patients on nutritional care, food, and eating. Using two data collection methods, focus groups with dietitians and individual patient interviews, allowed a multifaceted exploration of factors that can inform the nutritional care of this patient group. In the focus groups, dietitians discussed aspects they considered in the nutrition care of these patients in terms of (1) *Practical aspects*, (2) *Support and social aspects* and (3) *Feelings and emotions*. However, they highlighted that social and emotional aspects were not systematically assessed, monitored, or evaluated, applying instead measures such as body weight and nutritional intake to follow‐up and evaluate the nutritional care. The analysis of individual interviews with patients identified four themes: (1) *Ability to prepare and eat food*, (2) *Issues affecting food enjoyment*, (3) *Social eating and mealtimes* and (4) *Emotional relationship with food*. While there is some overlap between the results of the focus groups and the patient interviews, the patients focused predominantly on the negative impact of symptoms after stroke. These included the loss of food enjoyment, diminished social eating contexts, and the reduced emotional valence of food and mealtimes that had profoundly impacted their everyday life in relation to food and eating. There was, therefore, a discrepancy between the values of importance to patients and the aspects addressed by dietitians.

Dietitians select interventions that address nutritional problems to achieve desired nutritional care outcomes [14]. In nutrition intervention, setting SMART goals is advised, meaning that goals should be specific, measurable, achievable, relevant, and time‐framed [29, 30]. Dietitians are trained in setting goals that aim to improve the patient's nutritional intake and thereby their nutritional status. In line with this, the dietitians in this study reported evaluating nutritional care using outcomes of body weight and nutritional intake, which can be measured and quantified. However, the patients in this study were almost exclusively concerned with the aspects of food and eating that profoundly affected their everyday life. Not addressing such aspects can potentially impact the quality of nutritional care and thereby nutritional care outcomes.

Focusing only on energy intake and nutrient provision has been defined as ‘medicalising’ food and does not acknowledge the central role played by food in many human activities. Scrinis describes this as ‘nutritionism’, where people eat ‘nutrients’ for bodily health [31]. This idea neglects the social, cultural, religious, and traditional significance of food, as well as the pleasure derived from the smells and tastes experienced during eating and its importance for quality of life [32, 33]. While food consumption is essential to sustain life, eating and drinking are activities with significant social and emotional associations, from which people derive pleasure and well‐being [34]. If the pleasure of eating is lost, a person may limit their food intake and their participation in social gatherings involving food [32]. Stroke‐related impairments can have profound effects on many aspects of a person's life, which may result in physical, social, and emotional consequences, and greatly affect their sense of identity and well‐being [35, 36], as described by patients in this study. Such effects could lead to the social, cultural, and even religious aspects of eating and mealtimes being lost after a stroke.

Similar problems have been observed in patients with head and neck cancer, where a reduced ability to eat well was seen to impact quality of life with profound consequences for patients' physical and emotional health and well‐being [37]. Even in this group, an alteration in eating and a changed relationship with food was found to extend beyond nutritional aspects into areas of identity and social participation, and could be an enduring aspect of patients' lives. This is in line with the results from the present study. Focusing on weight and intake in the nutritional care of patients without considering the burden of altered eating and the negative impact of a changed relationship with food and eating on the social and emotional aspects of everyday may affect the outcomes of nutritional care of the patient group in this study.

The current lack of suitable tools limits the dietitians' ability to systematically monitor the aspects that patients in this study highlighted as profoundly affecting everyday life. The Altered Eating Framework was developed iteratively by Burges Watson et al. [25] in collaboration with survivors of head and neck cancer to identify how multiple domains of everyday life might be affected by an altered relationship with food after illness. The authors define ‘Altered Eating’ as ‘*a changed state of any combination of physical, emotional and social interactions with food and eating that has a negative impact on health and well‐being.’* This framework provides a systematic way of assessing how a person's eating experience might have changed after illness and the consequences of these changes, highlighting the multifactorial nature of a person's relationship to food.

The Altered Eating Framework could, therefore, help elucidate the results from our study on patients at risk of malnutrition after a stroke. The patients highlighted key areas where their altered relationship to food had impacted their everyday life. Several of these correspond to domains in the Altered Eating Framework, including physical, behavioural, social and emotional aspects. These aspects were, however, not addressed by the dietitians to the same extent. The Altered Eating Framework could, therefore, be used to support dietitians in systematically addressing such issues with these patients and thereby enhance the quality of their individualised nutritional care of patients after a stroke. Future studies based on the Altered Eating framework are needed to explore this further.

Healthcare today, both in Sweden and internationally, strives for a patient‐centred approach, where the partnership between patient and healthcare professional is seen as a prerequisite for good quality care and patient safety [16, 38, 39]. To be able to follow‐up the quality of interventions in healthcare from a patient perspective, patient‐reported outcome measurements (PROM) have received increased focus in recent years [40]. PROM refers to validated and standardized questionnaires that are developed and designed to measure patients’ self‐perceived health, function, and health‐related quality of life [41], and to clearly highlight the individual's needs and concerns [42]. However, there is a lack of suitable PROMs allowing the patient's evaluation of their nutritional treatment [22]. Since the Altered Eating Framework addresses dimensions of food and eating that can be meaningful to patients and which, if changed after illness, can impact their everyday life, it could provide the basis for the future development of a PROM to evaluate the nutritional care of patients at risk of malnutrition after a stroke from the patient's perspective.

## 5 Strengths and Limitations

A strength of this study is that the researchers all have broad knowledge of qualitative research and malnutrition; the first author also has clinical experience with patients treated for stroke. Data collection from both dietitians and patients, allowing a multifaceted analysis of the subject, is another strength. One strength of the focus groups with dietitians is the inclusion of representatives from both primary care and hospitals, where their experience from different settings promoted valuable discussions. Further, the focus groups allowed discussions specific to one diagnosis. The strengths of the patient interviews were that they occurred in the patient's own home, according to their preferences, and patients with communication difficulties were included, helped by close family members. A limitation was that all the participating dietitians and the moderator were colleagues, which may have influenced the discussions. However, the participants commented that they had not discussed this topic before and had gained new insights from the focus group discussions. Dietitians and patients were all recruited from one healthcare region, which is acceptable in qualitative studies, although including other healthcare regions may have contributed further insights. No non‐Swedish speaking patients were recruited, which limits transferability.

## 6 Conclusion

By exploring dietitians' and patients' perspectives, this study elucidates how the burden of altered eating and the negative impact of a changed relationship with food and eating reported by patients after a stroke are not fully captured by outcome measures currently available to dietitians. This is a key limitation in the current nutritional care of patients at risk of malnutrition after a stroke. While dietitians recognize the importance of the qualitative aspects of food and eating in everyday life, they lack appropriate tools to adequately assess and evaluate these.

The altered eating framework might provide the basis for the development of a patient‐reported outcome measure (PROM) to reflect patients' own perspectives relevant to their nutritional care after a stroke and enhance the quality of individualized nutritional care of patients at risk of malnutrition after a stroke.

## Author Contributions

All authors participated in the conception and design of the study, as well as the analysis and interpretation of the data. Jenny McGreevy performed the data collection and was responsible for drafting the manuscript. All authors critically revised the manuscript, and read and approved the final manuscript.

## Ethics Statement

Ethical approval was obtained from the Swedish Ethical Review Authority (Dnr 2019‐0256; amended 2022‐04286‐02).

## Conflicts of Interest

The authors declare no conflicts of interest.

## Peer Review

The peer review history for this article is available at https://www.webofscience.com/api/gateway/wos/peer-review/10.1111/jhn.70091.

## Transparency Declaration

The lead author affirms that this manuscript is an honest, accurate, and transparent account of the study being reported. The reporting of this study is compliant with SRQR guidelines [43]. No important aspects of the study have been omitted.

## Supporting information

Appendix A.

Appendix B.

## Data Availability

The data that support the findings of this study are available from the corresponding author upon reasonable request.
