# Supplementary material for: Patients' Voices and Dietitians' Perspectives on Meaningful Aspects in the Nutritional Care of Patients at Risk of Malnutrition After Stroke
Source: J Hum Nutr Diet. 2025 Jul 28;38(4):e70091. doi: 10.1111/jhn.70091 (PMC12304626; doi:10.1111/jhn.70091)
Supplement: Supplementary file 1 — Appendix A. [file JHN-38-0-s001.docx]

**Appendix A. Interview guide for the focus group discussions**

What aspects are important for you to assess in the nutritional care of a patient at risk of malnutrition after a stroke?

When you meet a patient at risk of malnutrition after a stroke and want to get an idea of the person’s situation, what is important for you to find out?

To what extent is the patient’s perspective considered in the nutrition intervention?

What aspects do you consider important to follow-up and evaluate in the nutritional care of these patients?

What is important for you to follow up next time?

What kind of goals do you set with the patient?

Are there any other aspects that you think are important to follow up? Why?

How do you assess, follow-up and evaluate these aspects?

What do you consider when evaluating the effect of nutritional care in these patients?

Do you see any challenges in following up and evaluating aspects of the nutritional care?

How do you assess if the patient’s nutritional problems have improved?

Is the patient involved in follow-up and evaluation?

How would you describe successful nutritional care of a patient at risk of malnutrition after a stroke?

What do you think is important for the patients in their nutritional care?

How do you think patients are affected by their nutritional problems and by the care and advice they receive? (quality of life, function in everyday life, health?)
