# Supplementary material for: Patients' Voices and Dietitians' Perspectives on Meaningful Aspects in the Nutritional Care of Patients at Risk of Malnutrition After Stroke
Source: J Hum Nutr Diet. 2025 Jul 28;38(4):e70091. doi: 10.1111/jhn.70091 (PMC12304626; doi:10.1111/jhn.70091)
Supplement: Supplementary file 2 — Appendix B. [file JHN-38-0-s002.docx]

**Appendix B. Interview guide for the patient interviews**

Can you tell me how you feel about meals, eating and drinking after your stroke?

Who cooks your food? Do you get any help with food and meals? If so, from who and in what way? Do you have any problems after your stroke that affect you when you eat and drink? Has anything changed regarding the planning of meals, shopping for food, or preparation/cooking of food and drink?

How were meals, eating and drinking for you before your stroke?

Did you have any problems with eating before your stroke? Do you have other conditions that affect your life, eating, or food and meal situation? Did you have any dietary restrictions before your stroke?

How do you feel that the changes to meals, eating and drinking after your stroke have affected your life?

Do you enjoy eating and drinking in the same way as you did before your stroke? Has anything changed after your stroke that affects your social life around food, if so in what way? Do you look forward to eating, drinking and mealtimes?
